# Validity of Lung Ultrasound: Is an Image Worth More Than a Thousand Sounds?

**DOI:** 10.3390/jcm10112292

**Published:** 2021-05-25

**Authors:** Cristina Ramos-Hernández, Maribel Botana-Rial, Marta Núñez-Fernández, Irene Lojo-Rodríguez, Cecilia Mouronte-Roibas, Ángel Salgado-Barreira, Alberto Ruano-Raviña, Alberto Fernández-Villar

**Affiliations:** 1 Hospital Alvaro Cunqueiro, Department of Pneumology, Neumo Vigo I + i, Institute of Health Research Galicia South (IISGS), 36312 Vigo, Spain; maria.isabel.botana.rial@sergas.es (M.B.-R.); marta.nunez.fernandez@sergas.es (M.N.-F.); irene.lojo.rodriguez@sergas.es (I.L.-R.); cecilia.mouronte.roibas@sergas.es (C.M.-R.); alberto.fernandez.villar@sergas.es (A.F.-V.); 2 Methodology and Statistics Unit, Galicia Sur Health Research Institute (IISGS), 36312 Vigo, Spain; angel.salgado.barreira@sergas.es; 3 Department of Preventive and Public Health, Faculty of Medicine, University of Santiago de Compostela, 15705 Santiago de Compostela, Spain; alberto.ruano@usc.es; 4 Consortium for Biomedical Research in Epidemiology and Public Health (CIBER en Epidemiología y Salud Pública, CIBERESP), 28029 Madrid, Spain; 5 Instituto de Investigaciones Sanitarias de Santiago de Compostela (IDIS), 15706 Santiago de Compostela, Spain

**Keywords:** LUS, ultrasound, lung auscultation

## Abstract

Introduction: There is debate as to whether lung-ultrasound (LUS) can replace lung-auscultation (LA) in the assessment of respiratory diseases. Methodology: The diagnostic validity, safety, and reliability of LA and LUS were analyzed in patients admitted in a pulmonary ward due to decompensated obstructive airway diseases, decompensated interstitial diseases, and pulmonary infections, in a prospective study. Standard formulas were used to calculate the diagnostic sensitivity, specificity, and accuracy. The interobserver agreement with respect to the LA and LUS findings was evaluated based on the Kappa coefficient (ᴋ). Results: A total of 115 patients were studied. LUS was more sensitive than the LA in evaluating pulmonary infections (93.59% vs. 77.02%; *p* = 0.001) and more specifically in the case of decompensated obstructive airway diseases (95.6% vs. 19.10%; *p* = 0.001). The diagnostic accuracy of LUS was also greater in the case of pulmonary infections (75.65% vs. 60.90%; *p* = 0.02). The sensitivity and specificity of the combination of LA and LUS was 95.95%, 50% in pulmonary infections, 76.19%, 100% in case of decompensated obstructive airway diseases, and (100%, 88.54%) in case of interstitial diseases. (ᴋ) was 0.71 for an A-pattern, 0.73 for pathological B-lines, 0.94 for condensations, 0.89 for pleural-effusion, 0.63 for wheezes, 0.38 for rhonchi, 0.68 for fine crackles, 0.18 for coarse crackles, and 0.29 for a normal LA. Conclusions: There is a greater interobserver agreement in the interpretation of LUS-findings compared to that of LA-noises, their combined use improves diagnostic performance in all diseases examined.

## 1. Introduction

One of the most widely used instruments in physical examinations is the stethoscope, which was developed in 1816 by French physician Laënnec [1]. This tool has become an important emblem of medicine and is a key element of the physical examination of patients with respiratory diseases [2,3]. However, the diagnostic validity and sensitivity of auscultatory symptoms is often too limited to allow for reaching an accurate diagnosis [2].

Thus, many authors consider that the stethoscope’s days may be numbered due to the development of new exploratory techniques [4]. Lung ultrasound (LUS) has become an essential complementary tool and its application in the Pulmonology practice is an unstoppable fact. From an etymological point of view, this tool is considered to be the real stethoscope, as stethos is a Greek prefix that refers to the thorax, while skopein, another Greek word, refers to the act of observing, and the only possible way to dynamically visualize the inside of the thorax at the patient’s bedside, without emitting ionizing radiation, is with LUS. Imaging techniques enable us to supplement the information obtained from the physical examination. For this reason, LUS is a key diagnostic tool in several Emergency and Critical Care service protocols [5,6,7]. However, in conventional hospital wards, the routine use of this technique is slow and often limited by factors such as the required learning curve, accessibility to an ultrasound system, or time constraints. Lung auscultation (LA) and LUS should be two key pillars in the comprehensive assessment of patients hospitalized for respiratory conditions. Still, despite the importance of these two diagnostic tests, the reproducibility of the signs evidenced with each one of them and whether their joint interpretation might improve the diagnostic results are still unclear.

On the premise that LUS should be considered the fifth fundamental pillar of physical examination, as an enhancer of the traditional examination, we propose this study with the following two objectives: first, to compare the diagnostic yield of LA and LUS for the diagnosis of different respiratory diseases for which patients are frequently admitted to Pulmonology wards and, second, to determine the interobserver variability when interpreting the different sounds detected during an LA and the images identified in an LUS, as this aspect has not yet been rigorously studied.

## 2. Materials and Methods

### 2.1. Study Design

A prospective study was carried out in the Pulmonology ward of a third-level hospital (Alvaro Cunqueiro Hospital in Vigo) between March 2019 and May 2019.

Inclusion criteria: Adult (>18 years) patients who had been admitted to this ward from an Emergency Care Department, due to a respiratory infection, decompensation of a previous obstructive disease, or interstitial involvement, were consecutively selected to form part of the study population.

Exclusion criteria: Patients whose clinical deterioration prevented them from sitting down to allow an LUS to be conducted, who had been admitted over 24 h earlier, with poor-quality sound recordings, and with suspected pulmonary thromboembolism or tumor, owing to the low expression of these conditions in the lung sounds, were excluded from the study. Patients who required admission to an intermediate monitoring unit or a critical care unit were also excluded from the study, as were those with viral pneumonia, as this was not a common diagnosis during the study period and prior to the 2019 coronavirus disease (COVID-19) pandemic.

All patients signed the informed consent form specific to this research study. The study was approved by the Galician Research Ethics Committee with code 2018/526.

### 2.2. Data Collection

#### 2.2.1. Demographic Variables and Final Diagnosis

Demographic variables, including the patients’ sex and age, were collected. The diagnosis used as a reference was that established by the responsible clinician at the time of discharge of each patient. The authors of this paper (ILR and CMR) reviewed each medical record retrospectively, three months after the patients’ discharge from the hospital, while being blinded to the LUS and LA findings, and determined the final diagnosis established by the responsible clinician at the time of discharge of each patient. Both authors jointly reviewed 115 medical records, and in cases of disagreement (12), they reached a consensus following an open discussion.

The following three categories were established for the analysis of the final diagnoses:

- Respiratory infection, including patients with bacterial pneumonia and bronchiectasis with superinfection.
- Decompensation of a previous obstructive disease, including patients with chronic obstructive pulmonary disease (COPD) and asthma.
- Interstitial involvement, including patients with a discharge diagnosis of interstitial lung disease (ILD) or congestive heart failure (CHF).

#### 2.2.2. Ultrasonographic Procedure and Variables

The LUS was performed by a pulmonologist (CRH) within the first 24 h of the patients’ admission to the hospital. This operator had over five years of experience in this technique and access to the patient’s medical records, but in no case was responsible for the patients’ care during their stay at the hospital. The study was performed with the patients in a seated position, examining an anterior area, two lateral areas, and a posterobasal area following the current recommendations [8]. A convex probe (2–5 Mhz) was used to assess the posterior and lateral areas, and a linear probe (5–10 Mhz) was used to examine the anterior intercostal spaces. A Sonosite M-Turbo ultrasound system (Bothell, Washington, USA) with a pulmonary preset was used in all cases. In the case of the areas examined with the convex probe, the examination was started at a depth of 12 cm, which was subsequently reduced to 6–8 cm after identifying the reference structures; in the case of the linear probe, the initial examination depth was approximately 6 cm, and could subsequently be reduced to 4 cm. The ultrasound was focused on the pleural line throughout the entire examination.

The LUS images were recorded at intervals of approximately 10 s per scanned area to allow their subsequent anonymized re-evaluation by the same pulmonologist who performed the examination (CRH) and a second observer (MBR) who did not have access to the patients’ medical records and was unaware of the findings described by the observer who performed the LUS. This second observer (MBR) had an accumulated experience of over two years in LUS and had not received specific training for the detection of the relevant alterations. Both operators recorded the presence or absence of the ultrasonographic signs described below in an anonymized log that was specifically designed for this research study.

Specific ultrasonographic patterns were defined based on the LUS findings.

The presence of more than two areas in each hemithorax with ≥3 B-lines per intercostal space was interpreted as an interstitial syndrome [9]. When these findings were associated with a heterogeneous distribution, a loss of pleural integrity, decreased pleural sliding, and/or the presence of subpleural abnormalities, the interstitial involvement was linked to a decompensated ILD. In contrast, when the distribution of the B-lines was homogeneous and associated with gravitational predominance, as well as with preservation of the pleural line and its sliding, the diagnosis was oriented toward a CHF [10].

The presence of focal B-lines, the detection of an echogenic tissue consolidation associated with the presence of a dynamic air bronchogram with an intact parietal pleura, or the combination of both of these findings in the LUS was considered compatible with an infection [9].

The presence of A-lines in both sides of the thorax was classified as decompensation of an underlying obstructive disease (asthma or COPD) [11].

#### 2.2.3. Pulmonary Auscultation Procedure and Variables

The LA was carried out after the LUS by the same operator (CRH), who explored the same areas that were examined with the LUS [8], using a 3M™ Littmann^®^ Model 3200 electronic stethoscope that enabled the recording of sounds for their subsequent analysis by another researcher (MNF). Each recording lasted approximately one minute and was performed at tidal volume. Two observers (CRH and MNF) boasting over 10 years of experience in the field of Pulmonology and who, therefore, did not require specific training to identify the respiratory sounds, anonymously classified the sounds captured in the recordings and only defined those that were evaluated.

The sounds detected during the LA were grouped into different categories corresponding to the definitions proposed by Loudon and Murphy [12] using the terminology recommended by the European Respiratory Society (ERS) [3], which allowed a diagnostic hypothesis to be established and the patients to be classified into the three main diagnostic groups described above: respiratory infection, decompensation of an underlying obstructive disease, and interstitial involvement.

Brief, discontinuous sounds were classified as crackles. These could be subclassified as coarse crackles if they had a high pitch indicative of the presence of secretions or intra-alveolar fluid, which oriented the diagnosis toward an alveolar-interstitial process or decompensation of an obstructive pathology. Fine crackles of shorter duration and a lower pitch, which are indicative of an abrupt alveolar opening caused by the presence of restrictive diseases, were linked to a decompensated ILD [2,3]. Continuous, low-pitched sounds, similar to snores, which may be caused by the airway fluttering or the movement of air through pulmonary secretions and that can be detected in alveolar-interstitial processes or decompensation of obstructive diseases, were classified as rhonchi [2,3]. Finally, sounds detected almost exclusively during the inspiratory phase and resembling “squeaks” were classified as wheezes. The presence of these sounds was indicative of a decrease in the airway caliber secondary to its inflammation or the presence of secretions, thus orienting the diagnosis toward a decompensation of an underlying obstructive disease or a respiratory infection [2,3]. An absence of these sounds on both sides of the thorax was considered to be normal auscultation.

### 2.3. Statistical Analysis

Qualitative variables were expressed as percentages and absolute frequencies, and quantitative variables were expressed as a median and interquartile range (95% confidence interval [CI]).

The sensitivity (Se), specificity (Sp), and diagnostic accuracy (DA) values of the LA and LUS were analyzed for each of the conditions studied, and their diagnostic performance was compared using the McNemar test to compare proportions.

The degree of interobserver agreement was represented using the Kappa (ᴋ) coefficient and its confidence interval (95% CI). A negative ᴋ value indicates that the degree of agreement is lower than what would have been expected by chance. Positive values of 0–0.2 indicate a poor agreement, values of 0.2–0.4 indicate a mild agreement, values of 0.4–0.6 indicate a moderate agreement, values of 0.6–0.8 indicate a good agreement, and those of 0.8–1 indicate a very good agreement [13]

A sample size estimation was performed for a diagnostic test with an Se of 80%, an Sp of 90%, and a DA greater than or equal to 18%, calculating a sample size of 110. The statistical analysis was performed with the IBM SPSS Statistics version 21 software (IBM Corp., Armonk, NY, USA).

## 3. Results

### 3.1. Sample Description

**Figure 1:**
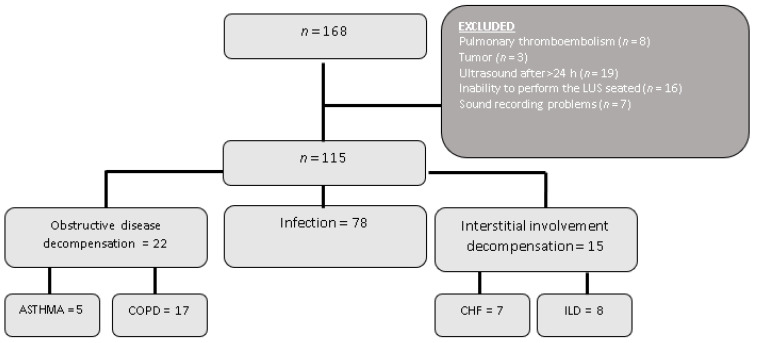
Flow chart. ILD = interstitial lung disease, CHF = Congestive Heart failure, COPD: Chronic obstructive pulmonary disease.

A total of 168 patients were included in the study. Of these, 53 were eventually excluded due to the reasons explained in Figure 1. Therefore, a final total of 115 patients were analyzed, with 76 of them (66%) being men with a mean age of 65.47 years (95% CI:62.8–68.4). In 22 (19.1%) patients, the final diagnosis was decompensation of their underlying obstructive disease, with 17 (77.3%) of these cases corresponding to a decompensated COPD and five (22.7%) to an asthma attack. A large proportion of the patients (78; 67.8%) were diagnosed with a respiratory infection, and 15 (13%) were diagnosed with a decompensated interstitial disease.

### 3.2. Pulmonary Auscultation and Lung Ultrasound Results

The different ultrasonographic signs and auscultatory sounds detected by the operator who performed the procedure (CRH) are listed in Table 1.

The diagnostic validity results of the LUS and LA are shown in Table 2.

Of the 15 patients included in the interstitial involvement group, eight were diagnosed with a decompensated ILD at discharge, and seven were diagnosed with a CHF. An independent analysis of the patients presenting with a decompensated ILD revealed significant differences favoring the LA with respect to the test’s Sp (97.19–17.65%; *p* = 0.00) and DA (94.78–21.73%; *p* = 0.00), but no significant differences in its Se (83.3–100%; *p* = 1.00).

The posterior power for the differences in sensitivity for infections is 82.6%, while for the differences in specificity and accuracy in obstruction it is 100%.

### 3.3. Interobserver Variability

The degree of interobserver agreement was good for the detection of an A-pattern (ᴋ = 0.71 (95% CI: 0.70–0.74)) and pathological B-lines (ᴋ = 0.73 (95% CI: 0.73–0.75)). This good agreement was maintained in the presence of focal B-lines (ᴋ = 0.73 (95% CI: 0.72–0.75)), with a very good agreement when the B-line involvement was diffused (ᴋ = 0.81 (95% CI: 0.81–0.83)). The agreement was also very good with respect to the detection of consolidations (ᴋ = 0.94 (95% CI: 0.93–0.95)) and pleural effusion (ᴋ = 0.89 (95% CI: 0.88–0.90)) by LUS (Table 3).

Regarding the auscultatory sounds, the interobserver agreement was good with respect to the detection of wheezes (ᴋ = 0.63 (95% CI: 0.61–0.65)) and fine crackles (ᴋ = 0.68 (95% CI: 0.66–0.70)), low for rhonchi (ᴋ = 0.38 (95% CI: 0.36–0.40)) and normal auscultations (ᴋ = 0.297 (95% CI: 0.27–0.31)), and poor for coarse crackles (ᴋ = 0.18 (95% CI: 0.16–0.20)) (Table 3).

## 4. Discussion

To our knowledge, this is the first study comparing the diagnostic contribution of lung sounds and ultrasonographic patterns in the different respiratory conditions representing the main causes of admission to a conventional hospital ward [14]. In addition, its results demonstrate a good degree of interobserver agreement in the interpretation of ultrasonographic signs, much greater than that seen in the interpretation of lung sounds. These findings are relevant, as they support the incorporation of LUS into conventional physical examination, thus involving a change in the approach toward hospitalized patients.

In general terms, the performance of LUS examinations is greater than that of LA for all conditions examined. Pulmonary auscultation only offered a greater diagnostic contribution in a subanalysis of a very small group of eight patients with ILD. These data coincide with those published in existing literature, where the detection of fine crackles has been reported in up to 60% of patients with ILD, even before the detection of radiological alterations [15].

The greater diagnostic performance of LUS was demonstrated in the examination of patients with pulmonary infection, with 93.59% of these cases exhibiting ultrasonographic alterations compatible with this diagnosis. As recommended in the consensus document [9], we defined an LUS pattern compatible with infection characterized by the presence of focal B-lines or consolidation. However, similar studies performed with pediatric populations show that, in an appropriate clinical context, the presence of diffused B-lines might be compatible with a multifocal infection [16]. In our case series, 27 (34.6%) patients in this group had an LUS with diffused B-lines. Thus, if this pattern were included as a diagnostic parameter for respiratory infection, the Se of the LUS would be 96.15%.

No differences were found between the Se of the tests in the group of patients presenting with obstructive disease, although a significantly greater Sp was detected in favor of the LUS. In the bedside lung ultrasound in emergency (BLUE) protocol [11], the presence of an A-pattern without associated findings of deep vein thrombosis was linked to a decompensated obstructive disease with a Se of 89% and aSp of 97%. In our case, we obtained similar Sp values but lower Se values, as 36.4% of the patients had an LUS with pathological B-lines or consolidations instead of an A-pattern. We believe that this can be explained by the fact that, in our study, we used the diagnosis established at the time of discharge as the reference diagnosis, which in this case was decompensated asthma/COPD, while the diagnosis could have possibly corresponded to a respiratory infection that triggered such decompensation and was not viewed in the X-rays. In fact, when we retrospectively reviewed the group of patients presenting with LUS alterations, we discovered that all of them had received antibiotic treatment. The Global Initiative for Chronic Obstructive Lung Disease (GOLD) guidelines [17] recommend the use of antibiotics in patients with COPD exacerbations based on clinical criteria. Some studies have also examined certain biochemical markers such as the C-reactive protein (CRP) or procalcitonin, but their results have been controversial. Lung ultrasound might provide additional information on which patients with a COPD exacerbation require antibiotic treatment. In the case of asthma, the Global Initiative for Asthma (GINA) guidelines [18] do not recommend antibiotic treatment during an asthma attack. Nevertheless, in our sample, at least two patients who had been admitted to the hospital for an asthma attack and had normal chest X-ray findings presented with LUS consolidations that required antibiotic treatment.

The Se results obtained in our study for the LUS are similar to those published for critically ill patients (97% in the case of those with consolidations and 95% in those with alveolar-interstitial syndromes) [19]. However, in our study, we observed a greater diagnostic performance for the LA. In critically ill patients, LA has demonstrated an Se of 55% for the diagnosis of alveolar-interstitial syndromes and of 36% for that of consolidations [19]. We believe that the greater diagnostic performance of LA in our sample could be explained by the fact that we examined a larger area of the thorax by approaching the posterior lung fields.

A prerequisite for assessing the reproducibility of a diagnostic test and accurately inferring results is to have a good interobserver agreement. Although the interpretation of LUS images requires specific training and experience, because ultrasonographic signs are well categorized and easy to identify, operator dependence is minimal, as evidenced by the high interobserver agreement found in the interpretation of the LUSs performed in our study (Table 3). These results are similar to those published to date, where interobserver agreement for the assessment of an A-pattern [20] is ᴋ = 0.90, for an alveolo-interstitial pattern [20,21] is ᴋ = 0.74–0.91), and for a consolidation [20,22] is ᴋ = 0.76–0.78. The interobserver agreement with respect to the lung sounds was significantly lower (Table 3), although this coincides with the data reported in the available literature, where the highest degree of interobserver agreement is obtained for wheezes and fine crackles, followed by rhonchi and normal auscultation [23,24,25]. The only result obtained in our study that did not coincide with the data reported in the available literature is the interobserver agreement with respect to normal auscultation, as the ᴋ obtained in our study was 0.297 and those published in other studies range between 0.41 and 0.46 [21,23]. This can be due to the fact that the lung sound recordings might have also captured skin friction sounds, which could have led to an incorrect interpretation. An aspect that should be kept in mind regarding the evaluation of these lung sounds is that we focused on traditional pulmonary auscultation through a sound-recording stethoscope. There are new devices [26,27] that allow for analyzing the acoustic and physiological features of lung sounds and that could improve diagnostic performance and interobserver agreement. However, these devices require the use of post-processing software that reduces bedside decision-making but lengthens the time required to reach a diagnosis as compared to LA and LUS.

Our study has some strengths, such as the anonymized assessment of the LUS and LA findings for a second time based on the recordings. In addition, it covered a heterogeneous sample with different pulmonary diseases. This ensured an independent interpretation of the auscultatory sounds and ultrasonographic signs while avoiding selection or measurement biases. Nevertheless, our study also has certain limitations, such as the fact that conducting a global assessment of the usefulness of LUS among patients hospitalized in a Pulmonology ward limits the number of cases of certain diseases. Hence, these results should be validated in multicenter studies in order to be extrapolated to the general population. However, this study does not aim to establish comparisons between the individualized contributions for each disease, but rather to share the findings described with other clinicians, as we believe they are sufficient to support the use of LUS as a complement to the traditional physical examination. Moreover, the intensity of the sound recordings was not graduated. In the appropriate context, the intensity of the sounds can aid in the diagnosis of the disease, as they are more intense in the presence of deep breathing and attenuated in the presence of pleural effusion, pneumonia, decreased ventilation in obstructive diseases, or a lack of patient cooperation [26]. Given that the second observer was unaware of the conditions under which the LA was performed, we decided not to include this aspect in our study. In addition, some authors have questioned its clinical usefulness [28]. We also include as a limitation that we could expect the coexistence of signs and symptoms of two or more comorbidities. In such a case, the interpretation of LUS and LA could be masked by the overlapping of the presence of characteristic signs of different diseases.

## 5. Conclusions

We did not intend to generate a debate comparing auscultation with ultrasound examination based on these results; instead, we sought to highlight the importance of integrating LUS as the fifth fundamental pillar of bedside physical examination, as it increases the overall Se and Sp in all diseases analyzed. Neither LUS nor LA are laboratory tests, but rather a component of the physical examination whose usefulness depends on their adequate correlation with the available clinical information. However, the evidence supporting LUS is increasingly solid for the diseases that most frequently lead to admission to the Pulmonology ward [14]. For all the above reasons, we believe that it is time to take a step forward and incorporate LUS into the traditional physical examination.

## Figures and Tables

**Table 1:** Lung Ultrasound and Pulmonary Auscultation Characteristics per Subgroup.

Discharge Diagnosis	LUS Findings *	LA Findings *
Respiratory infection (*n* = 78)	A-pattern 3 (3.8%) Pathological B-lines 66 (84.6%) Focal 39 (50%) Diffuse 27 (34.6%) Consolidation 35 (44.8%) Pleural effusion 21 (26.9%) 9 (11.5%) >3 intercostal spaces	Rhonchi 28 (35.9%) Crackles Fine 1 (1.2%) Coarse 24 (30.7%) Wheezes 14 (17.9%) Normal 13 (45.5%)
Underlying obstructive disease (*n* = 22)	A-pattern 14 (63.6%) Pathological B-lines 7 (31.8%) Focal 6 (27.3%) Diffuse 1 (4.5%) Consolidation 5 (22.7%) Pleural effusion 1 (4.5%) <1 intercostal space	Rhonchi 5 (22.7%) Crackles Fine 0 Coarse 3 (16.6%) Wheezes 5 (22.7%) Normal 10 (45.4%)
Interstitial involvement (*n*= 15) ILD (*n* = 8)	A-pattern 0 Pathological B-lines 8 (100%) Focal 1 (12.5%) Diffuse 7 (87.5%) Hypoechoic artifacts effacing the pleura 3 (37.5%) Pleural thickening 5 (62.5%) Consolidation 0 Pleural effusion 0	Rhonchi 1 (12.5%) Crackles 6 (75%) Fine 5 Coarse 1 Wheezes 2 (13.3%) Normal 0
CHF (*n* = 7)	A-pattern 0 Pathological B-lines 7 (100%) Focal 1 (14.3%) Diffuse 6 (87.7%) Consolidation 0 Hypoechoic artifacts effacing the pleura 0 Pleural thickening 0 Pleural effusion 3 (42.9%) Only 1 >3 intercostal spaces	Rhonchi 2 (28.6%) Crackles 4 (57.1%) Fine 2 Coarse 2 Wheezes 1 (14.3%) Normal 0

* LUS = Lung Ultrasound, LA = Lung Auscultation.

**Table 2:** Combined diagnostic performance of the pulmonary auscultation and lung ultrasound.

Discharge Diagnosis		LUS	LA	*p*	LUS and/or LA
		Focal B-Lines and/or Consolidation	Rhonchi and/or Crackles and/or Wheezes		
Respiratory infection (*n* = 78)	Sensitivity	93.59%	77.02%	*p* = 0.00	95.95%
	Specificity	37.88%	27.77%	*p* = 0.58	50%
	Diagnostic accuracy	76.65%	60.90%	*p* = 0.08	
		A-pattern	Rhonchi and/or crackles and/or wheezes	*p*	LUS and/or LA
Underlying obstructive disease (*n* = 22)	Sensitivity	63.63%	52.38%	*p* = 0.72	76.19%
	Specificity	95.60%	19.10%	*p* = 0.00	100%
	Diagnostic accuracy	89.51%	25.40%	*p* = 0.00	
		Diffuse B-lines	Crackles	*p*	LUS and/or LA
Interstitial involvement (*n* = 15)	Sensitivity	86.66%	66.67%	*p* = 0.45	100%
	Specificity	73%	76.04%	*p* = 0.57	88.54%
	Diagnostic accuracy	74.75%	74.77%	*p* = 0.31	

**Table 3:** Interobserver variability.

Lung Ultrasound	
A-pattern	ᴋ = 0.71 (95% CI: 0.70–0.74)
Pathological B-lines	ᴋ = 0.73 (95% CI: 0.73–0.75)
Focal B-lines	ᴋ = 0.73 (95% CI: 0.72–0.75)
Diffuse B-lines	ᴋ = 0.81 (95% CI: 0.81–0.83)
Consolidation	ᴋ = 0.94 (95% CI: 0.93–0.95)
Pleural effusion	ᴋ = 0.89 (95% CI: 0.88–0.90)
**Lung Auscultation**	
Wheezes	ᴋ = 0.63 (95% CI: 0.61–0.65)
Fine crackles	ᴋ = 0.68 (95% CI: 0.66–0.70)
Coarse crackles	ᴋ = 0.18 (95% CI: 0.16–0.20)
Rhonchi	ᴋ = 0.38 (95% CI: 0.36–0.40)
Normal	ᴋ = 0.29 (95% CI: 0.27–0.31)

## Data Availability

Data available on request due to restrictions (e.g., privacy or ethical).
